# Development of Lipid-Based Gastroretentive Delivery System for Gentian Extract by Double Emulsion–Melt Dispersion Technique

**DOI:** 10.3390/pharmaceutics13122095

**Published:** 2021-12-06

**Authors:** Jelena Mudrić, Katarina Šavikin, Ljiljana Đekić, Stefan Pavlović, Ivana Kurćubić, Svetlana Ibrić, Jelena Đuriš

**Affiliations:** 1 Institute for Medicinal Plants Research “Dr. Josif Pančić”, Tadeuša Košćuška 1, 11000 Belgrade, Serbia; ksavikin@mocbilja.rs; 2 Department of Pharmaceutical Technology and Cosmetology, Faculty of Pharmacy, University of Belgrade, Vojvode Stepe 450, 11221 Belgrade, Serbia; ljiljanadjekic@gmail.com (L.Đ.); ivana.kurcubic@pharmacy.bg.ac.rs (I.K.); svetlana.ibric@pharmacy.bg.ac.rs (S.I.); jelena.djuris@pharmacy.bg.ac.rs (J.Đ.); 3 Institute of Chemistry, Technology, and Metallurgy-National Institute for the Republic of Serbia, University of Belgrade, Njegoševa 12, 11001 Belgrade, Serbia; stefan.pavlovic@ihtm.bg.ac.rs

**Keywords:** solid lipid microparticles, SLM, gastroretentive system, Gelucire 43/01, Gelucire 39/01, Sylysia 350, gentiopicroside, double (W/O/W) emulsion, mucoadhesion, direct compression, biphasic release

## Abstract

Gentian (*Gentiana lutea* L., Gentianaceae) root extract (GRE) is used for the treatment of gastrointestinal disorders. However, its bioactive potential is limited in conventional forms due to the low bioavailability and short elimination half-life of the dominant bioactive compound, gentiopicroside. The aim of study was to encapsulate GRE in the lipid-based gastroretentive delivery system that could provide high yield and encapsulation efficiency, as well as the biphasic release of gentiopicroside from the tablets obtained by direct compression. Solid lipid microparticles (SLM) loaded with GRE were prepared by freeze-drying double (W/O/W) emulsions, which were obtained by a multiple emulsion–melt dispersion technique, with GRE as the inner water phase, Gelucire^®^ 39/01 or 43/01, as lipid components, with or without the addition of porous silica (Sylysia^®^ 350) in the outer water phase. Formulated SLM powders were examined by SEM and mercury intrusion porosimetry, as well as by determination of yield, encapsulation efficiency, and flow properties. Furthermore, in vitro dissolution of gentiopicroside, the size of the dispersed systems, mechanical properties, and mucoadhesion of tablets obtained by direct compression were investigated. The results have revealed that SLM with the macroporous structure were formulated, and, consequently, the powders floated immediately in the acidic medium. Formulation with porous silica (Sylysia^®^ 350) and Gelucire^®^ 43/01 as a solid lipid was characterized with the high yield end encapsulation efficiency. Furthermore, the mucoadhesive properties of tablets obtained by direct compression of that formulation, as well as the biphasic release of gentiopicroside, presence of nanoassociates in dissolution medium, and optimal mechanical properties indicated that a promising lipid-based gastroretentive system for GRE was developed.

## 1. Introduction

Yellow gentian (*Gentiana lutea* L., Gentianaceae) root is officially listed in the European and many national Pharmacopoeias [1]. Gentian root extract is used for the treatment of numerous gastrointestinal disorders, such as loss of appetite, functional dyspepsia, and liver dysfunction [2,3]. The main bioactive compounds of gentian root extract are secoiridoids, particularly gentiopicroside as a dominant compound, with manifested gastroprotective, choleretic, hepatoprotective, and anti-inflammatory activities [4]. However, the bioactive potential of gentian extract is limited in conventional forms due to the low bioavailability and short elimination half-life of gentiopicroside [5]. In addition, a local gastric effect of gentian extract was reported [6], indicating that prolonged release of gentiopicroside in the stomach could be beneficial.

Consequently, in order to improve the bioavailability and effectiveness, as well as patient compliance, it is necessary to enable the release of an initial, effective dose of gentian extract, followed by further sustained release at the place of action (stomach). This would allow improved absorption of the gentian extract active compounds. Since the effectiveness of gastroretentive delivery systems is influenced by numerous factors (gastric fluid level, presence of food, and gastric contents), the combination of different gastroretention strategies such as floating and mucoadhesion is desired [7] in order to increase the residence time of the gentian extract in the stomach. Lately, solid lipid microparticles have been applied as a carrier for the bioactive compound site-specific delivery [8]. Furthermore, this type of carrier provides good in vivo tolerability, adequate stability, and increased bioavailability, with quite low production costs and feasibility of large-scale production [9]. Lipid-based particles are commonly used for the encapsulation of lipophilic active compounds, whereas the inefficient incorporation of hydrophilic compounds, such as gentiopicroside, has been considered as their main disadvantage. However, the double emulsion (W/O/W) method could be used to overcome this limitation by incorporating hydrophilic active compounds or extracts in the inner water phase [10,11]. It is known that the formulation of double emulsions could be a challenging task because of their low thermodynamic stability [12]. Therefore, the selection of lipids, as well as lipophilic and hydrophilic emulsifiers as essential components of W/O/W emulsions are the critical steps in the formulation of solid lipid microparticles for stomach-specific delivery, i.e., lipid-based gastroretentive system. Solid lipids, Gelucire^®^ 43/01 and Gelucire^®^ 39/01, are promising materials in the formulation of gastroretentive delivery systems due to their high lipophilicity and low density, whereas their low melting temperatures (approximately 43 °C and 39 °C, respectively) are considered as favorable, to prevent thermal degradation of the bioactive compounds from the gentian extract during the W/O/W emulsion processing [13,14]. Furthermore, their triglyceride-based nature promotes contact with intestinal membranes, increases the solubilization and formation of triglyceride-rich chylomicrons, and reduces gastric emptying, and, thereby, improves the absorption of bioactive compounds [15]. Lipophilic emulsifiers such as polyglycerol ester of polyricinoleic acid (PGPR) and Spans^®^ (sorbitan esters) are required to stabilize the W/O interface, whereas emulsifiers with higher HLB values, such as Tweens^®^ (polysorbates), lecithin and proteins, are used as stabilizers of the O/W interface [10]. Generally, formulations containing lipids and surfactants in contact with the aqueous media could undergo the process of dissolution and molecular self-assembly, i.e., they spontaneously interact to form organized nanostructures such as micelles, mixed micelles, or more complex organized nanostructures. In addition, the bioavailability of encapsulated bioactive compounds increases as the lipid droplet size decreases, indicating that the size of dispersed systems could influence the effectiveness of the final product.

However, the fact that dosage forms resulting from W/O/W emulsions are usually liquids presents a drawback from the patient and industry point of view. Furthermore, the literature data support the use of gentian extract in solid dosage forms [6]. Therefore, it could be vital to transform liquid W/O/W emulsion into a powder form. Moreover, directly compressible powders are preferred in the pharmaceutical industry, while it is possible to produce tablets by using the direct compression method, which is simple, environmentally friendly (solvent/heat-free), and time/cost-effective. It is hypothesized that the properties of a lipid-based gastroretentive delivery system loaded with gentian extract could be improved by the incorporation of highly porous micronized silica (Sylysia^®^ 350), which has been widely employed as a powdering agent and carrier for liquid formulations [16].

Therefore, the objective of this study was to develop a lipid-based gastroretentive delivery system loaded with the gentian extract that could provide high yield and encapsulation efficiency, as well as the biphasic release of gentiopicroside from tablets obtained by direct compression of powder obtained by the freeze-drying of double emulsions with gentian extract as the inner water phase and Gelucire^®^ 39/01 or 43/01, as lipid components, without the addition of organic solvents.

## 2. Materials and Methods

### 2.1. Materials

Gentian (*Gentiana lutea*, Gentianaceae) roots were purchased from the Institute for Medicinal Plants Research “Dr. Josif Pančić” (Belgrade, Serbia). Gelucire^®^ 43/01 and Gelucire^®^ 39/01 were obtained as gift samples from Gattefossé^®^ (Saint-Priest, France). Polyglycerol polyricinoleate was obtained as a gift sample from Palsgaard^®^ (Juelsminde, Denmark). Trehalose dihydrate was purchased from TCI Chemicals (Tokyo, Japan). Span^®^ 80 (sorbitan oleate), Tween^®^ 80 (polysorbate 80), Tween^®^ 20 (polysorbate 20), and sodium chloride were obtained from Merck Co., Germany, while sodium alginate was purchased from Fisher Scientific, soybean lecithin was obtained from Serva Chemical Co. (Heidelberg, Germany), Sylysia^®^ 350 was obtained from Fuji Silysia chemical ltd. (Kasugai Aichi, Japan). For the mucoadhesion evaluation, the mucin from a porcine stomach, Type II (Sigma-Aldrich, Shanghai, China) was used. All other chemicals were of analytical grade, including orthophosphoric acid (Sigma-Aldrich Chemie GmbH, Münich, Germany), acetonitrile (Merck, Darmstadt, Germany). Ultra-pure water was prepared using a Milli-Q purification system (Millipore, Guyancourt, France). Gentiopicroside standard was obtained from ChromaDex (Los Angeles, CA, USA).

### 2.2. Gentian Extract Preparation

Gentian (*Gentiana lutea*, Gentianaceae) root extraction was performed in conical percolator by standard percolation procedure with ethanol as extraction solvent (50%, *v*/*v*) while solid to solvent ratio was 1:2 (g/mL). Consequently, ethanol was evaporated by using a rotary vacuum evaporator (IKA^®^ RV 05, Staufen, Germany), and the obtained liquid extract was filtrated and stored in a refrigerator in the dark bottle.

### 2.3. Preparation of Double W/O/W Emulsion

**Figure 1:**
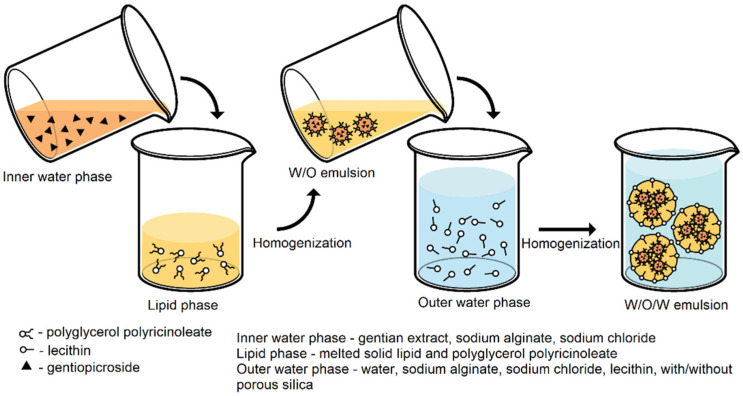
Process scheme.

Double emulsions were prepared according to the multiple (double) emulsion–melt dispersion technique as summarized in Figure 1. Firstly, preheated water phase (liquid gentian extract, sodium alginate, and sodium chloride) was added dropwise to the melted lipid phase (solid lipid: Gelucire^®^ 43/01 or Gelucire^®^ 39/01 and lipophilic emulsifier) according to the composition represented in Table 1, with constant stirring on a magnetic stirrer at 500 rpm (IKA^®^ RCT standard, Staufen, Germany) at temperature (5–10 °C) above the lipid melting point. In the preliminary study, primary emulsion (W/O) was formulated with Span^®^ 80 or PGPR as lipophilic emulsifiers. The obtained primary emulsion (W/O) was homogenized at 15,000 rpm for 3 min by using a high-shear homogenizer (Ultra-Turrax, IKA^®^, Staufen, Germany), while the temperature was constantly in the range presented in Table 1.

In the second step, hot (temperature indicated in Table 1) primary emulsion (W/O) with PGPR as lipophilic emulsifier was dispersed in the outer water phase containing a hydrophilic emulsifier, sodium alginate, sodium chloride, trehalose with or without Sylysia^®^ 350 (Table 2). The temperatures of emulsions with Gelucire^®^ 39/01 as solid lipid in the primary emulsions were in the range 44–49 °C, whereas the temperatures of emulsions with Gelucire^®^ 43/01 were in the range 48–53 °C. In the preliminary study, Tween^®^ 80, Tween^®^ 20, or lecithin were used as hydrophilic emulsifiers. Trehalose was used in all formulations as a cryoprotectant. Subsequently, emulsion (W/O/W) was homogenized at 3000 rpm for 4.5 min by a high-shear homogenizer (Ultra-Turrax, IKA^®^, Staufen, Germany). Furthermore, double emulsion (W/O/W) was continually stirred by a laboratory mixer (Heidolph RZR 2020, Heidolph Elektro GmbH & Co., KG, Kelheim, Germany) until the temperature of the prepared W/O/W emulsion was approximately 25 °C.

### 2.4. Characterization of Double Emulsions

#### 2.4.1. Conductometric Analysis

The conductivity of prepared samples was measured by using a conductivity meter (Radiometer, Copenhagen, Denmark) 24 h after preparation at 22 ± 2 °C. Each sample was analyzed in triplicate.

#### 2.4.2. Centrifugation Test

The stability of prepared samples (A–D) with PGPR as lipophilic emulsifier and lecithin as hydrophilic emulsifier was determined by centrifugation of double emulsion and by measuring the supernatant volume (mL). The samples were taken into centrifuge tubes and centrifuged (Hermle Z206A, Labortechnik GmbH, Wehingen, Germany) for 15 min at 5000 rpm at 22 ± 2 °C. Each sample was analyzed in triplicate.

#### 2.4.3. Microscopic Analysis

Microscopic analysis of the investigated emulsions after carefully diluting samples with purified water was conducted by using an Optical microscope (Olympus^®^ BX 41, Olympus Optical Co., Tokyo, Japan).

### 2.5. Preparation of Solid Lipid Microparticles

Prepared double emulsions (A–D) with PGPR as lipophilic emulsifier and lecithin as hydrophilic emulsifier were lyophilized by the in-house method developed in the PVP-Centre for Lyophilization (Valjevo, Serbia). Furthermore, liquid gentian extract obtained by the procedure described in Section 2.2 was dried under the same conditions in order to prepare gentian extract powder, which was compared with developed SLM formulations. Obtained freeze-dried materials were ground and stored in a desiccator.

### 2.6. Characterization of Solid Lipid Microparticles

#### 2.6.1. Scanning Electron Microscopy

The morphology of dry gentian extract and solid lipid microparticles powders was estimated by using JEOL JSM-6390LV scanning electron microscope (JEOL USA, Inc., Peabody, MA, USA). Prior to the analysis, samples were coated with gold for 100 s under 30 mA ion current on BALTEC SCD 005 sputter coater (Balzers, Switzerland).

#### 2.6.2. Determination of the Encapsulation Efficiency

Accurately weighed samples of solid lipid microparticles (80–100 mg) were placed in volumetric flasks with 2 mL of hot (70–80 °C) purified water. Samples in the flasks were heated in a water bath at 70 °C and sonicated for 30 min to melt the lipid component. Afterwards, the samples were diluted to 5 mL with hot purified water and filtered (0.45 μm cellulose acetate membrane filters). The concentration of gentiopicroside in samples was determined by high-performance liquid chromatography (Section 2.6.3). Gentiopicroside encapsulation efficiency (*EE*) was calculated according to Equation (1) [14]. Each sample was analyzed in triplicate and the results were presented as mean ± standard deviation. The differences among samples were tested by one-way ANOVA and subsequently estimated by Tukey’s post hoc test. Statistical analysis was performed using the MS Office Excel v. 2010.

(1) $$EE=\frac{Actual\;quantity\;of\;gentiopicroside\;entrapped\;in\;particles}{Theoretical\;quantity\;of\;gentiopicroside\;}\times 100$$

#### 2.6.3. High-Performance Liquid Chromatography

Analyses were performed on Agilent 1200 RR HPLC instrument (Agilent, Waldbronn, Germany), on a reverse-phase Zorbax SB-C18 (Agilent, Waldbronn, Germany) analytical column (150 mm × 4.6 mm i.d.; 5 μm particle size) according to the previously described procedure [17]. Identification of the marker compound (gentiopicroside) was achieved by comparing their UV spectra and retention time with those from authentic standards, while the concentration was determined from the peak areas by using the equation for linear regression obtained from calibration curves (correlation coefficient was 0.998).

#### 2.6.4. Determination of Yield

The yield was determined as the percentage of obtained microparticles mass (*A*) with respect to the expected mass of total solids used for their production (*B*). The solid content of liquid materials was determined after measuring dry residue on a moisture analyzer (Mettler Toledo HB43-S, Melbourne, Australia). Each sample was analyzed in triplicate. The differences among samples were tested by one-way ANOVA and subsequently estimated by Tukey’s post hoc test. Statistical analysis was performed using the MS Office Excel v. 2010.

(2) $$Yield\;\left(\%\right)=\frac{A}{B}\times 100$$

#### 2.6.5. Mercury Intrusion Porosimetry

Mercury intrusion porosimetry measurements were performed in the fully automated conventional apparatus Carlo Erba Porosimeter 2000 (pressure range: 0.1–200 MPa; pores with a diameter within 7.5 nm and 15,000 nm). The acquisition of the analysis data was performed using the Milestone Software 200. The measurements were conducted in two consecutive runs to remove potential suspicions of the presence of interparticle (voids) and intraparticle spaces, but also to indicate the dominant type of pores. The samples were evacuated for 2 h in a dilatometer placed in the Macropores Unit 120.

#### 2.6.6. Flow Properties Evaluation

Flowability of the investigated powders was evaluated by calculating the Hausner ratio and the Compressibility index [1], according to Equations (3) and (4), where *V_0_* is unsettled apparent volume and *Vf* is the final tapped volume determined after tapping the powder by the tapped density tester (Stampfvolumeter, STAV 2003, Jel, Ludwigshafen, Germany). Each sample was analyzed in triplicate and the results were presented as mean ± standard deviation.

(3) $$Hausner\;ratio=\frac{Vo}{Vf}$$

(4) $$Compressibility\;index=\frac{Vo-Vf}{Vf}\times 100\;$$

#### 2.6.7. Tablets Preparation and Determination of Mechanical Properties

Tablets were prepared by direct compression of powdered solid lipid microparticles. Tableting properties were examined by using a benchtop single-punch tablet press Gamlen D series (Gamlen Tableting Limited, London, UK) equipped with a flat punch (6 mm). The samples (approximately 100 mg of powder) were compressed at different loads (30 to 150 kg), while compaction speed (60 mm/min) and dwell time (0.08 s) were the same for each tablet sample. No lubricant was added before or during compression. For each phase of the tableting process (compression, detachment, and ejection) a force-displacement curve was generated by the instrument software, and the data were used to calculate the tensile strength, Equation (5); detachment stress, Equation (6); ejection stress, Equation (7) [18]. Tablet hardness and diameter were measured by using a hardness tester (Erweka^®^ TBH 125D, Erweka, Heusenstamm, Germany). Tablet thickness was estimated after the tablet ejection by the digital caliper. Each sample, compressed under defined load, was analyzed in triplicate and the results were presented as mean ± standard deviation.

(5) $$\sigma \;\left(MPa\right)=\frac{2\times F}{\pi \times R\times t}$$

(6) $$Detachment\;stress\;\left(MPa\right)=\frac{D\times 4\;}{R^{2}\times \pi }$$

(7) $$Ejection\;stress\;\left(MPa\right)=\frac{E}{R\times \pi \times t}$$

Tablet tensile strength—*σ*; force applied for tablet breaking—*F* (*N*); the tablet diameter—*R* (mm); the tablet thickness—*t* (mm); the maximal force of detachment phase—*D* (*N*); the maximal force of ejection phase—*E* (N).

#### 2.6.8. In Vitro Gentiopicroside Dissolution Testing

Dissolution of gentiopicroside (marker compound in the gentian extract) from formulated solid lipid microparticles (in the form of tablets) and the freeze-dried gentian extract (in the form of powder) was tested. Gentian extract powder and solid lipid microparticles in the form of tablets (A–D) were tested using USP IV (Flow-through cell, CE7 smart, Sotax, Aesch, Switzerland) apparatus, at 37 ± 0.5 °C, with a flow rate of 8 mL/min, during 6 h, while dissolution medium was 0.1 M HCl (100 mL). Samples were withdrawn and filtrated at 15, 45, 90, 150, 240, 360 min and immediately replaced with a fresh medium. Equal volumes of filtrated samples (2.5 mL) were combined and used as a pooled sample of investigated formulations according to the procedure for Botanical dosage forms described in the United States Pharmacopeial Convention [19]. The concentration of dissolved gentiopicroside in those samples was determined by high-performance liquid chromatography (Section 2.6.3).

Furthermore, the model-independent index described by Moore and Flanner [20], known as the similarity factor (*f_2_*) was used in order to statistically demonstrate the differences between the dissolution profiles of investigated samples, according to Equation (8). A value of *f_2_* lower than 65 implies that the profiles are significantly different, while the value of *f_2_* in the range from 65 to 100 indicates similarity between the profiles of over 95% [21].

(8) $$f_{2}=50\times log\left\{{\left[1+\frac{1}{n}\overset{t}{\underset{n=1}{\sum }}{\left(R_{t}-T_{t}\right)}^{2}\right]}^{-0.5}\times 100\right\}$$

where *n* is the number of dissolution sampling times, *R_t_* and *T_t_* are the active compound (gentiopicroside) release percentage at each time for the reference and test sample, respectively.

#### 2.6.9. Kinetic Modeling of Gentiopicroside Release

Gentiopicroside release kinetics were analyzed by using the data of a six-hour dissolution profile of each formulation and fitting into zero-order, first-order, Higuchi, and Korsmeyer–Peppas models. The highest correlation coefficient (r^2^) value is indicative of the actual model of the release.

#### 2.6.10. Assessment of Dispersibility during In Vitro Dissolution

In order to assess the dispersibility of the solid lipid microparticles (in the form of tablets), the size of the dispersed nanostructures in the dissolution medium (0.1 M HCl) was measured in the samples collected during the in vitro gentiopicroside dissolution testing of formulations A–D, at the first time point (after 15 min) and the last (after 6 h), using photon correlation spectroscopy (PCS). The size distribution was characterized by Zetasizer Nano ZS90 (Malvern Instruments, Malvern, UK) with the integrated He-Ne laser at 633 nm and scattered light detector at 90 °C. The measurements were performed at 20 ± 0.1 °C.

#### 2.6.11. Mucoadhesion Evaluation

Mucoadhesion of tablets composed of formulated solid lipid microparticles was determined as a force required to separate the investigated tablet (5 samples for each formulation) from the mucin disk. Texture Analyzer Shimadzu EZ-LX (Shimadzu Corporation, Kyoto, Japan), with a 5 kg load cell and 10 mm aluminum cylindrical probe, was used to measure the force of adhesion. Before the analysis, each tablet was glued to the upper probe, whereas mucin disc was attached to the surface of the platform by cyanoacrylate glue. Mucin discs were prepared by compression of commercially available raw gastric porcine mucin (250 mg) using a die of 13 mm in diameter. Afterward, the mucin disk was poured in 0.1 M HCl (2.5 mL) heated up to 37 °C for 4 min. A force of 0.5 N was applied on the tablet for 60 s to ensure intimate contact between the tablet and the mucin disc, where the pre-test speed was 1 mm/s, the test speed of 0.5 mm/s, and the post-test speed of 0.5 mm/s. The obtained force-time curves were utilized to determine the force of adhesion, i.e., the force required to separate the tablet from the mucin disc, as an indicator of tablet mucoadhesion. The study was performed in 5 replicates for each formulation and the results were presented as mean ± standard deviation. The differences among samples were tested by one-way ANOVA and subsequently estimated by Tukey’s post hoc test. Statistical analysis was performed using MS Office Excel v. 2010.

## 3. Results and Discussion

### 3.1. Double Emulsions Development

**Figure 2:**
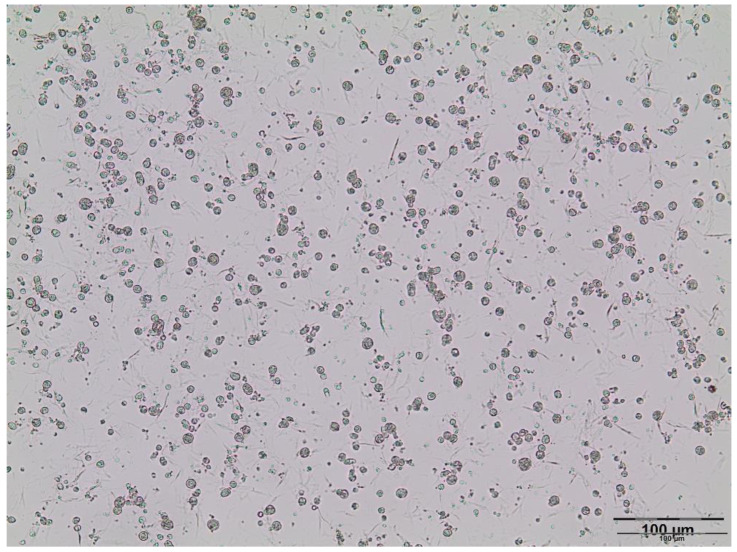
Photomicrograph of double (W/O/W) emulsion D.

Critical parameters in the development of a stable W/O/W emulsion is the selection of the appropriate lipophilic (W/O) and hydrophilic (O/W) emulsifiers. Span^®^ 80 (5%) as a lipophilic low molecular emulsifier was first used for stabilization of the W/O emulsion. However, formulations were unstable, and rapid phase separation was detected (approximately upon 5 min). On the other hand, W/O emulsions with a lipophilic polymeric emulsifier, PGPR (5%), were stable during one month of storage at room temperature. This result was in accordance with the previous findings, where PGPR was able to interact more effectively with the oil phase than Span^®^ 80 and, consequently, to prevent droplets’ coalescence due to higher hydrophobicity [10]. Therefore, PGPR was chosen as an appropriate emulsifier for the stabilization of primary (W/O) emulsion. Furthermore, it is reported that the addition of biopolymers such as sodium alginate to the inner water phase provides the formation of a viscoelastic barrier, thus preventing coalescence, as a result of the interaction between polysaccharide and the lipophilic emulsifier [10]. Additionally, the primary emulsion thermodynamic stability could be improved by incorporating the electrolytes such as sodium chloride in the inner water phase since collision frequency and droplet sizes are reduced in that way due to a decrease in attractive forces between water droplets and reduction in interfacial tension [22,23]. For that reason, sodium alginate (1.3%) and sodium chloride (0.05 M) were added to the inner water phase. Furthermore, to ensure uniform osmotic pressure in the primary (W/O) and secondary (W/O/W) emulsion, and to avoid diffusion between the inner and outer phase, sodium chloride and sodium alginate were also added to the outer water phase. In order to choose the optimal hydrophilic emulsifier, double emulsion with Tween^®^ 80, Tween^®^ 20, and lecithin were prepared. Immediately after processing, in the emulsions with Tween^®^ 80 and Tween^®^ 20, phase separation was evident. On the other hand, double emulsions with lecithin (samples A–D) were stable during a week of refrigerated storage. Emulsions A–D were yellow and homogenous, and there were no changes in consistency and homogeneity, and phase separation was not detected after the centrifugation test. The conductivity of investigated emulsions A (4.03 ± 0.24 µS/cm), B (4.28 ± 0.31 µS/cm), C (4.53 ± 0.18 µS/cm), and D (4.77 ± 0.13 µS/cm) indicates that the water phase was the external phase of the obtained emulsion. It is known that high conductivity suggests an O/W or W/O/W emulsion, while a low conductivity (<1 µS/cm) could indicate a W/O or O/W/O emulsion [24]. According to the microscopic analysis, the investigated emulsions with Gelucire^®^ 43/01 or Gelucire^®^ 39/01, as well as with or without Sylysia^®^ 350 were characterized as double (W/O/W) emulsions with a complex inner structure inside oil droplets, i.e., microsphere (C type)-type double emulsions (Figure 2). It is known that the double emulsion type is significantly influenced by the type of hydrophilic surfactant and the concentration of hydrophobic surfactant. Furthermore, emulsions with a higher concentration of the hydrophobic surfactant are generally considered as microsphere-type double emulsions, which are more stable emulsions with higher encapsulation efficiency [25,26].

### 3.2. Solid Lipid Microparticles Characterization

#### 3.2.1. Morphology

**Figure 3:**
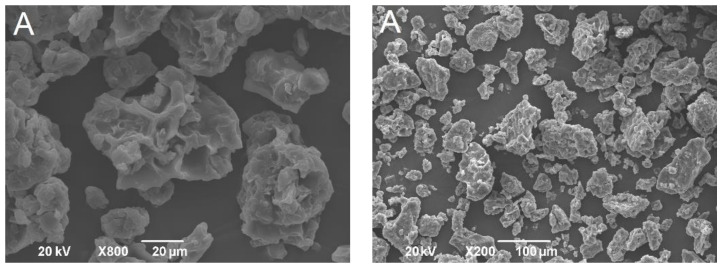

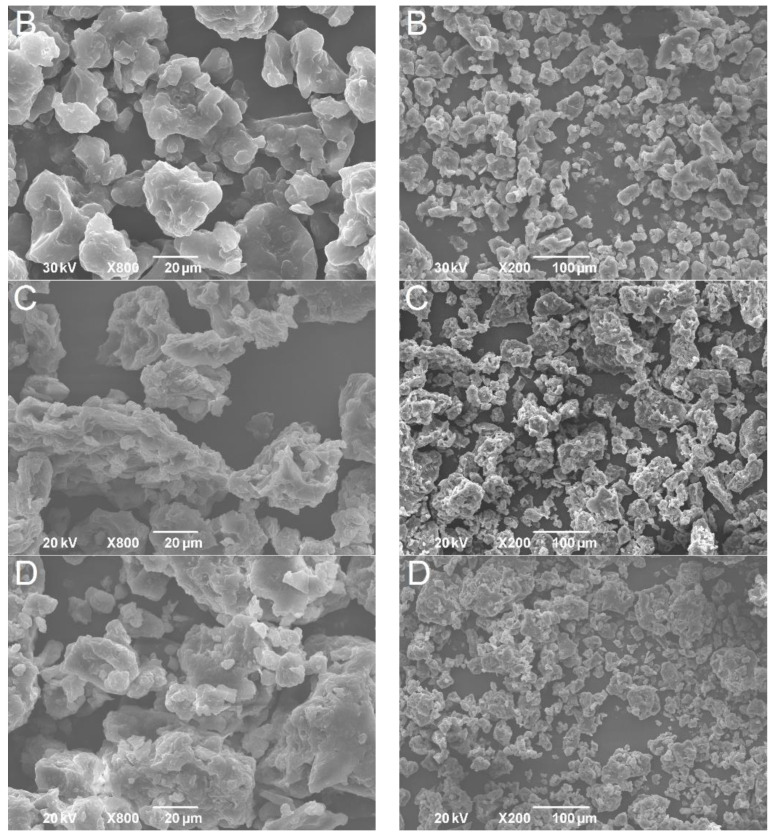
SEM pictures of solid lipid microparticles (formulation (**A**)-first row; formulation (**B**)-second row; formulation (**C**)-third row; formulation (**D**)-fourth row).

**Figure 4:**
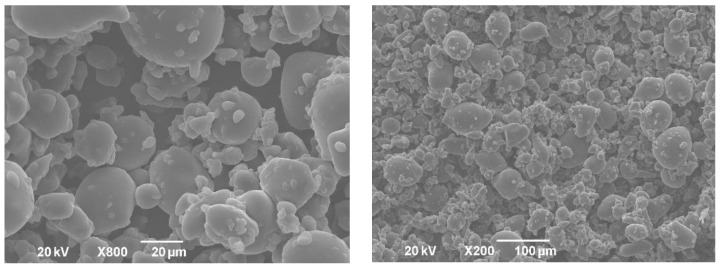
SEM pictures of dry gentian extract powder.

The powders obtained after the freeze-drying of double emulsions were homogenous and yellow. The shape and size of formulated powders were analyzed by SEM. Obtained solid lipid particles (Figure 3) were characterized with amorphous structures, similar to flakes of varying size, likely as a result of the samples grinding. This shape is common for microparticles obtained by the freeze-drying process [27]. The particle size (diameter), even with some aggregation, was under 1000 µm, indicating that solid lipid microparticles were developed. Higher magnification of the samples revealed the porous structure of microparticles. It has been reported previously that pores in microparticles are formed due to the sublimation of ice crystals during freeze-drying [28]. On the other hand, dry gentian extract particles were spherical but pores were not detected (Figure 4).

#### 3.2.2. Yield and Encapsulation Efficiency

The yield of solid lipid microparticles was very high for all investigated formulations (formulations A–D), indicating the appropriate materials and effective method were selected (Table 3). Furthermore, the encapsulation efficiency of all investigated formulations was very high, above 95% of the initial amount of gentiopicroside was incorporated in the solid lipid microparticles (Table 3). Consequently, solid lipid microparticles with Gelucire^®^ 43/01, as well as with Gelucire^®^ 39/01 as a lipid component, were considered as optimal for encapsulation of the hydrophilic bioactive compounds such as gentiopicroside. It was suggested recently that high encapsulation efficiency in solid lipid (nano)particles with Gelucires as lipid components could be achieved due to their heterogeneous nature (saturated polyglycolized glycerides consisting of mono-, di-, and tri-glycerides and mono- and di-fatty acid esters of polyethylene glycol), which leads to more structural imperfections and the formation of more space in the lattice that enables incorporation of the active compound [29]. Previously, it was reported that carnauba wax microparticles, prepared by a multiple emulsion–melt dispersion technique, were characterized with high encapsulation efficiency (above 80%) in the case of a water-soluble drug, i.e., pseudoephedrine hydrochloride [11]. Consequently, it was confirmed that the multiple emulsion–melt dispersion technique was a suitable method for encapsulation of the hydrophilic active substances into solid lipid microparticles without the use of an organic solvent. On the other hand, a moderately modified multiple emulsion–melt dispersion procedure was used by Peres et al. for encapsulation of the hydrophilic drug into solid lipid nanoparticles, but the encapsulation efficiency was lower (63%) [30]. Moreover, it is known that gentiopicroside is quite unstable [31]. Despite this, the results are indicating that there was no degradation of gentiopicroside, during the production of solid lipid microparticles. It was reported that gentiopicrin and oleanolic acid were loaded simultaneously into nanostructured lipid carriers, while lower total encapsulation efficiency (48.34%) was determined [32]. Therefore, the high encapsulation efficiency of water-soluble (bioactive compounds) gentian extract is achieved by the selection of a suitable W/O/W emulsion formulation and process parameters.

#### 3.2.3. Porosity

The porosity of investigated powders, i.e., dry gentian extract and solid lipid microparticles loaded with gentian extract, was examined by mercury intrusion porosimetry. Properties of the obtained powders are shown in Table 4. Dry gentian extract exhibited the highest bulk density (1.22 g/cm^3^), while the bulk density of formulated solid lipid microparticles was lower (0.93–1.03 g/cm^3^). Moreover, solid lipid microparticle powders (A–D) floated on the simulated gastric fluid (0.1 M HCl) surface, while the gentian extract was dissolved immediately after the contact with the medium, and flotation was not realized. Generally, powders with a density lower than the density of the gastric fluid (1.004 g/cm^3^) could be considered as floating delivery systems [7]. The lower bulk density of solid lipid microparticles could be attributed to the porosity of the obtained powders and the composition of particles. It was reported that Gelucire^®^ 43/01 (true density 0.0856 g/cm^3^) and Gelucire^®^ 39/01 are lipid materials with low density [13,14]. The porosity of solid lipid microparticles in the first run was in the range from 24.7% to 32.8%, whereas the porosity determined in the second run was lower and varied within the range of 16.2% and 21.3%. The second run was conducted to remove the eventual influence of interparticle and intraparticle effect, which can result in apparent porosity. It was evident that the mercury intruded volume in the second run decreased, which can indicate, firstly, the interparticle porosity according to the nature of materials, and, secondly, the presence of a special pore type in the literature known as ink bottle-shaped [33]. Such shaped pores cannot discharge during the extrusion phase in the first run, wherein they remain occupied by mercury and are unavailable for the next intrusion cycle. According to the SEM micrographs, it could be realized that the porosity of solid lipid microparticles is attributed to the surface porosity, whereas the reduction in porosity in the second run could be attributed to the interparticle porosity and presence of ink bottle-shaped pores. On the other hand, the porosity of dry gentian extract in the second run was significantly lower (3.1%) than in the first run (20.2%), indicating the intensive presence of interparticle porosity characteristic of powder samples. The non-porous nature of dry gentian extract was confirmed by SEM micrographs (Figure 4). It is reported that porous microparticles can remain buoyant in gastric content, resulting in prolonged gastric residence time [34]. Therefore, this result indicates that formulated porous solid lipid microparticles are promising gastroretentive delivery systems. Furthermore, the average pore size of formulated solid lipid microparticles corresponds to large pore diameter, suggesting that macroporous materials are obtained [34]. The highest porosity after the first run was observed in the case of formulations with 1% of Sylysia^®^ 350 (B and D), whereas formulation D was characterized with the highest porosity in the second run and the lowest bulk density (0.93 g/cm^3^). This result could be related to the high porosity of Sylysia^®^ 350 [35].

#### 3.2.4. Flowability

A continuous and uniform flow of the powders is required during the production of tablets, as well as other solid dosage forms, in order to provide accurate dosing. Therefore, Carr’s index (Compressibility index) and the Hausner ratio of all powder formulations were investigated. The results, presented in Table 5, revealed that formulated powders (A–D) were characterized with fair to excellent flowability according to the European Pharmacopoeia [1]. On the other hand, the dry gentian extract showed poorer properties, possibly as a result of the amorphous structure and unfavorable physicochemical and mechanical properties of dry herbal extracts that could cause the poor flowability of powders and compressibility in the tablet compression process [36]. Therefore, this result indicates that encapsulation of the extract in the double emulsion prior to freeze-drying improves the processability of this material.

Generally, Sylysia^®^ 350 is added externally in the tablet’s formulation in order to improve powder flowability [37]. However, according to the obtained results, formulations A and C (without Sylysia^®^ 350) were considered superior in comparison to the same formulations with 1% of Sylysia^®^ 350 (B and D). Furthermore, it was reported that the addition of colloidal silicon dioxide (Aerosil^®^) to the solid dispersion of Gelucire^®^ 44/14 and curcumin influenced the angle of repose significantly [38], indicating that the flowability of powders was reduced, similar to our results.

#### 3.2.5. Mechanical Properties 

**Figure 5:**
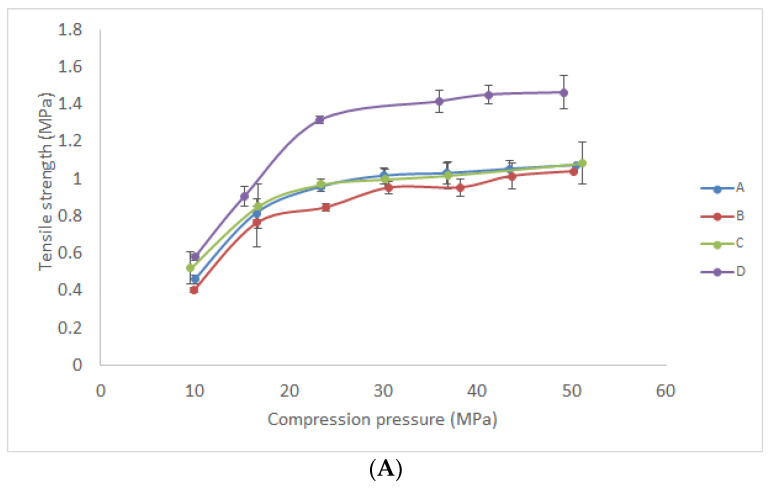

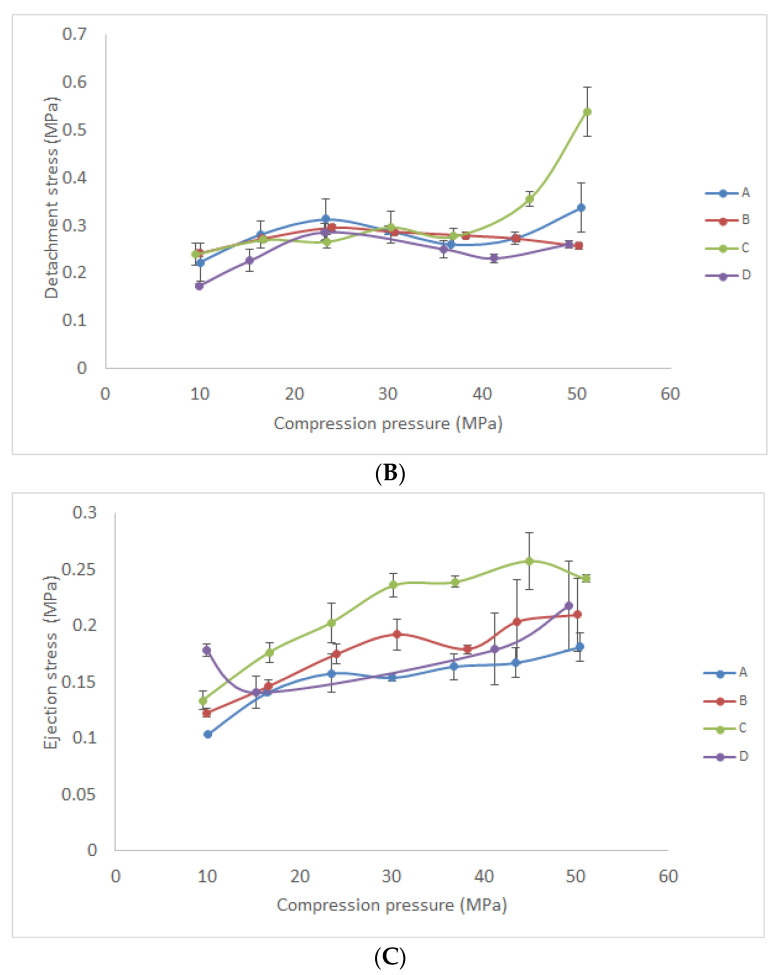
Tableting properties of the investigated materials compressed under different compression pressures: (**A**) tensile strength; (**B**) detachment stress; (**C**) ejection stress.

It was necessary to examine the mechanical properties of formulated powders in order to provide material with the optimal characteristics for tablet production, transport, and application. Previously, Gelucire^®^ 43/01 and Gelucire^®^ 39/01 were employed as release-retarding agents and melting binders in tablet formulation, but the mechanical properties of those powders were not investigated [39,40]. Results revealed that the tensile strength of tablets manufactured under low compression pressure (approximately 10–50 MPa) was in the range from 0.4 to 1.5 MPa (Figure 5a). Tablets compressed under the pressure below 10 MPa were too fragile, and tablet hardness was not measurable by the applied hardness tester. In general, the desired tablet tensile strength should be higher than 1 MPa [41]. The tensile strength of tablets manufactured by direct compression of powders A–C was similar under the same compression pressure, while tablets manufactured from powder D were characterized with higher tensile strength.

Furthermore, ejection (Figure 5c) and detachment stress (Figure 5b) were investigated as parameters that are used to describe the lubricating properties of tableting material. Generally, these parameters are affected by the type of tableting material and compression pressure, as well as by the finish and chemical nature of the tablet punch. Examined tablets of all investigated formulations were characterized with very low ejection stress (0.09 to 0.26 MPa) in the considered compression pressure range. According to the literature, ejection stress should be lower than 3 MPa to prevent failure during tablet production [18,42]. Consequently, this result indicated that there was no need for the addition of a lubricant in any of the investigated formulations. Moreover, the detachment stress of formulated tablets was examined since high detachment stress could be an indicator of interaction (adhesion or stickiness) between the powder and die surface [18]. According to the previous reports, detachment stress could be lower or higher in comparison with ejection stress. Drastically higher detachment than ejection stress could reveal significant interaction of the powder and die material. In the Figure 5b,c it is shown that similar or slightly higher detachment than ejection stress was determined in the case of formulations B and D. On the other hand, in the case of formulations A and C, an increase in detachment stress was evident under a pressure greater than 50 MPa. This result suggests that formulations with Sylysia^®^ 350 were characterized with lower detachment stress and minor differences between detachment and ejection stress. Consequently, tablets manufactured by the compression of powder D under low pressure (23–50 MPa) were characterized with sufficient tensile strength (≥1 MPa) and low detachment and ejection stress. This result suggests that this formulation could be used in the production of tablets based on solid lipid microparticles loaded with gentian extract by direct compression.

#### 3.2.6. Mucoadhesivity Evaluation

Mucoadhesive properties of solid dosage forms are commonly evaluated by the application of the texture analyzer measurements, whereas mucoadhesive strength is usually measured as the maximum force needed for detaching the formulation from the mucous membrane or substrate [43]. All tablets prepared by the direct compression of solid lipid microparticles loaded with gentian extract showed mucoadhesive properties. Investigated formulations (A–D) were characterized by the average force of adhesion in a range from 1.73 to 2.46 N, as presented in Table 6, and there was no significant difference (*p* = 0.506) between formulations. The force of adhesion of 0.94 N was reported in the case of the optimized gastroretentive delivery system for allopurinol based on a combined approach of mucoadhesion and floating, and this system has enabled gastroretention in vivo, using the albino rabbits model [44]. The high force of adhesion of formulated tablets could be attributed to the presence of sodium alginate since it was described as an anionic mucoadhesive polymer with higher mucoadhesive strength than polymers such as polystyrene, carboxymethylcellulose, and poly (lactic acid) [45]. Moreover, it was observed that Gelucire^®^ 43/01 and 39/01 have no adhesive properties but function as a structural agent that helps in the formation of a more robust gel and, consequently, better adhesion of tablets with hydroxypropyl methylcellulose and chitosan as hydrophilic polymers [21]. Therefore, the developed tablets are considered as mucoadhesive systems, and it could be expected that these systems are able to prolong retention time at the targeted place (i.e., stomach) and to provide intimate contact between the dosage form and the gastric mucosa.

#### 3.2.7. In Vitro Gentiopicroside Release

**Figure 6:**
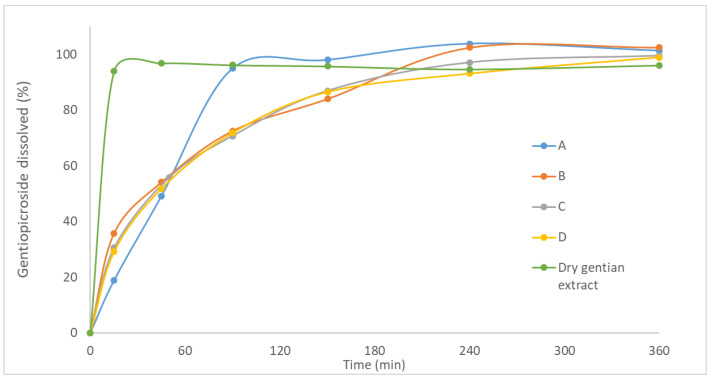
The dissolution profile of gentiopicroside from investigated formulations (A–D) of tablets and dry gentian extract powder.

Dissolution profiles of gentiopicroside from solid lipid microparticles loaded with gentian extract in the form of tablets, as well as from the dry gentian extract powder are presented in Figure 6. The rapid dissolution of gentiopicroside from dry gentian extract was evident. It was shown that after 15 min, gentiopicroside was completely dissolved. This result is expected due to the high solubility of gentiopicroside. On the other hand, dissolution profiles of gentiopicroside from solid lipid microparticles loaded with gentian extract (A–D) were significantly different in comparison to the dry gentian extract. Formulations A–D were characterized with the biphasic release, exhibiting burst release of gentiopicroside in the first 45 min and slower release in the second phase (until 6 h). Therefore, it could be expected that the initial (effective) dose of extract would be dissolved in the first 45 min, while the sustained release in the next 5–6 h could provide the prolonged effect of the gentian extract in the stomach, due to the previously explained mucoadhesive properties and floating ability of investigated tablets during the in vitro dissolution testing. This type of release was described previously in the case of solid lipid microparticles [9]. The sustained release of gentiopicroside indicated the effective incorporation of gentian extract inside the particle matrix. Furthermore, it could be suggested that the composition of solid lipid microparticles influenced the release rate since Gelucire^®^ 39/01 and 43/01 were used as release-retarding hydrophobic excipients in the formulation of sustained floating multiparticulate delivery systems [13,40]. Additionally, under acidic conditions, sodium alginate is transformed into a hydrogel that controls the release of active compounds [46]. On the other hand, rapid release in the first phase could be influenced by the porous structure and presence of gentiopicroside on the surface of solid lipid microparticles. It was reported that the porous structure of microparticles could provide a shorter pathway for the movement of bioactive compounds and water molecules [14].

The dissolution profiles of gentiopicroside from formulations (A and B) with the Gelucire^®^ 39/01 as a lipid component were different (Figure 6), indicating that the presence of Sylysia^®^ 350 (1%) in the formulation B decreased the dissolution rate of gentiopicroside. This result is consistent with previous findings, where the addition of colloidal silicon dioxide resulted in a slower release of curcumin from self-microemulsifying drug delivery systems incorporated in gastroretentive alginate-based composite sponges [46]. Furthermore, the slower release of propranolol from the physical mixture of Eudragit RS and colloidal silicon dioxide suggested that the binding between colloidal silicon dioxide and the active compound managed to reduce the dissolution rate [47]. On the other hand, the dissolution profiles of formulations C and D were similar, indicating that the addition of Sylysia^®^ 350 influenced the dissolution rate of gentiopicroside only in the case of lipid with a lower melting point (Gelucire^®^ 39/01).

Furthermore, according to the obtained results (*f_2_* < 65), dissolution profiles of formulations A and C, as well as dissolution profiles of formulations B and D were not similar. A slower release rate was accomplished in the formulation with Gelucire^®^ 43/01 than in the formulation with Gelucire^®^ 39/01, indicating that the selection of the lipid component could have a significant influence on the dissolution rate of gentiopicroside. It was reported previously that the softening of the Gelucire^®^ is a crucial factor for controlling the active compound release and that release rate was higher in granules with Gelucire^®^ 39/01 than in granules with Gelucire^®^ 43/01 [21].

Finally, the correlation coefficients (r^2^) obtained after the adjustment of dissolution test experimental data to different mathematical models (zero-order, first-order, Higuchi, and Korsmeyer–Peppas) are presented in Table 7. The Korsmeyer–Peppas model was considered to be the most suitable model for describing the release kinetics of gentiopicroside from all formulations of solid lipid microparticles loaded with gentian extract in the form of tablets. Furthermore, all formulations had release exponent (*n*) below 0.45, suggesting that gentiopicroside was released from tablets dominantly by following the Fickian diffusion mechanism. This result is consistent with previous findings, where the Korsmeyer–Peppas model was applied to characterize the release mechanism of clopidogrel bisulfate from prepared Gelucire^®^ 43/01 microcarriers, while the *n* value indicated that the Fickian diffusion mechanism was dominant [14].

#### 3.2.8. Dispersibility during In Vitro Dissolution

**Figure 7:**
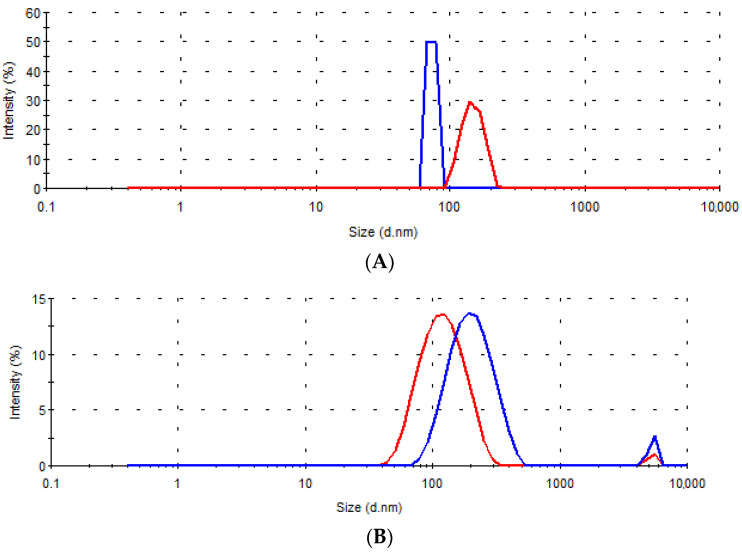

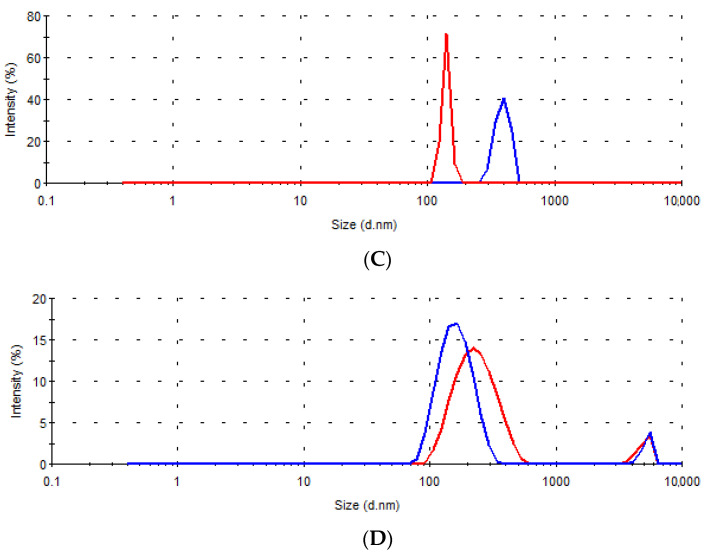
Size distribution by intensity in the dissolution media samples collected after 15 min (––) and 6 h (---) for the tablets (**A**–**D**).

Figure 7 shows the size distributions of dispersions obtained and sampled during the in vitro dissolution testing for the tested samples A–D, and in Table 8 the size diameters corresponding to each peak and their intensity are summarized.

In all tested samples, after only 15 min, nanoassociates of size less than 500 nm were observed. In addition, at this time point, in the medium in which the tablets of samples B and D were tested, the presence of microparticles of about 5 μm in size was detected, which most likely represents the silica (Sylysia^®^ 350) added to these two formulations. The average Sylysia^®^ 350 particle size, according to the manufacturer’s specification is 3.9 μm [48], and increased silica particle size could be the consequence of liquid W/O/W emulsion absorption [16].

Differences in the dispersibility of solid lipid microparticles were most pronounced at the very beginning of the dissolution test. The smallest size of nanoassociates was present in the dissolution medium samples of tablets A, which could indicate faster dispersion of microparticles from tablets A and, consequently, coincide with the observed higher rate of gentiopicroside release compared to other formulations. The nanoassociates were present in the dissolution medium samples even after 6 h for all tested formulations (Figure 7, Table 8). The observed presence of the surfactant and lipid-based nanoassociates in the medium corresponding to an acidic environment in the stomach may be advantageous for a prospective increase in the absorption of the bioactive compound in vivo [49].

## 4. Conclusions

An effective method for the encapsulation of gentian extract into solid lipid microparticles was developed by freeze-drying double (W/O/W) emulsions with solid lipid, i.e., Gelucire^®^ 39/01 or 43/01, with polyglycerol polyricinoleate as lipophilic emulsifier and lecithin as a hydrophilic emulsifier. Moreover, this organic solvent-free method could be highly beneficial for the encapsulation of other liquid extracts or bioactive compounds in an advanced carrier, which could enable modified release.

All investigated solid lipid microparticle formulations were characterized with very high yield (above 90%) and gentiopicroside (marker compound) encapsulation efficiency (above 95%), demonstrating that the developed technique was suitable for the encapsulation of hydrophilic bioactive compounds into solid lipid microparticles without the use of organic solvent. Furthermore, SEM analysis and mercury intrusion porosimetry revealed that solid lipid microparticles with the macroporous structure were formulated, and, consequently, the powders floated immediately in the acidic medium (0.1 M HCl). On the other hand, freeze-dried gentian extract was a non-porous powder, which was immediately dissolved in the same medium. Formulated powders have shown fair to excellent flowability according to the European Pharmacopoeia, while dry gentian extract has shown poorer properties, indicating that the encapsulation of an extract in the double emulsion before freeze-drying has improved the processability of this material. During the direct compression of formulated powders, it was evident that the addition of lubricant was not necessary. All tablets prepared by the direct compression of solid lipid microparticles loaded with gentian extract have shown mucoadhesive properties. Finally, in vitro dissolution tests have shown that the biphasic release of gentiopicroside from formulated solid lipid microparticles in the form of tablets was achieved. Therefore, the prolonged effect of the gentian extract in the stomach could be expected, due to mucoadhesive properties and the floating ability of the investigated formulations, while the presence of the surfactant and lipid-based nanoassociates could suggest the increase in the absorption of bioactive compounds in vivo.

In addition, formulation D with porous silica (Sylysia^®^ 350) and Gelucire^®^ 43/01 as a solid lipid was characterized with the hight yield end encapsulation efficiency, as well as with the highest porosity and the lowest bulk density (0.93 g/cm^3^) among the investigated formulations. The flowability of this formulation was good according to the European Pharmacopoeia, while the detachment, ejection stress, and tensile strength of manufactured tablets were optimal, suggesting that this formulation could be used in the production of tablets by direct compression. Furthermore, the mucoadhesive properties of the formulation D in the form of tablets, as well as the biphasic release of gentiopicroside and presence of nanoassociates indicate that a promising lipid-based gastroretentive carrier for the site-specific delivery of gentian extract was developed. In further studies, it would be essential to investigate the bioavailability of developed formulation.

## Figures and Tables

**Table 1:** A–D: The primary (W/O) emulsion composition.

	A	B	C	D
Gentian extract (%)	66.5	66.5	66.5	66.5
Sodium alginate (%)	1.3	1.3	1.3	1.3
Sodium chloride (M)	0.05	0.05	0.05	0.05
Gelucire^®^ 39/01 (%) *	26.9	26.9	/	/
Gelucire^®^ 43/01 (%) **	/	/	26.9	26.9
Lipophilic emulsifier (%) ***	5.0	5.0	5.0	5.0

* Emulsions were prepared at 44–49 °C; ** Emulsions were prepared at 48–53 °C; *** Span^®^ 80 or polyglycerol polyricinoleate.

**Table 2:** A–D: Double (W/O/W) emulsion composition.

	A	B	C	D
Primary emulsion with PGPR (%) *	19.8	19.8	19.8	19.8
Sodium alginate (%)	2	2	2	2
Sodium chloride (%)	0.05	0.2	0.2	0.2
Hydrophilic emulsifier (%) **	1.6	1.6	1.6	1.6
Trehalose (%)	7.9	7.9	7.9	7.9
Sylysia^®^ 350 (%)	/	1.0	/	1.0
Purified water to (%)	100.0	100.0	100.0	100.0

* Corresponding primary emulsion was marked with the same letter in Table 1; PGPR-polyglycerol polyricinoleate. ** Tween^®^ 80, Tween^®^ 20, or lecithin.

**Table 3:** Encapsulation efficiency of prepared solid lipid microparticles loaded with gentian extract (A–D).

Sample	A	B	C	D
EE (%) *,**	98.92 ± 1.06 a	103.02 ± 0.15 ab	98.77 ± 4.28 a	104.32 ± 0.16 b
Yield (%) **	92.05 ± 1.48 a	95.17 ± 1.03 a	91.85 ± 2.03 a	93.57 ± 1.87 a

* EE—gentiopicroside encapsulation efficiency; ** Means followed by the same letters (a or b) in the same row are not significantly different according to ANOVA (Tukey’s test), *p* ≤ 0.05.

**Table 4:** Average pore diameter, bulk density, and porosity of powders A–D (solid lipid microparticles) and dry gentian extract.

Sample	Run	D_av_ (µm)	BD (g/cm^3^)	P (%)
A	I	9.78	1.03	24.7
	II	9.78	1.03	16.2
B	I	9.78	0.95	32.8
	II	9.00	0.95	16.7
C	I	6.29	0.93	29.9
	II	9.78	0.93	17.3
D	I	9.0	0.93	32.4
	II	9.0	0.93	21.3
Dry gentian extract	I	9.8	1.22	20.2
	II	0.01	1.22	3.1

D_av_—Pore diameter average; BD—Bulk density; P—porosity.

**Table 5:** Flowability of obtained solid lipid microparticles (A–D) and dry gentian extract.

Sample	Hausner Ratio	Carr Index (%)	Flowability
A	1.13 ± 0.04	11.84 ± 3.11	good
B	1.23 ± 0.02	18.67 ± 1.26	fair
C	1.10 ± 0.02	9.73 ± 1.48	excellent
D	1.12 ± 0.03	10.64 ± 2.18	good
Dry gentian extract	1.28 ± 0.08	21.95 ± 5.26	passable

**Table 6:** The force of adhesion of tablets manufactured by direct compression of powders A–D.

Sample	A	B	C	D
The force of adhesion (N) *	1.73 ± 0.66 a	2.02 ± 0.46 a	2.08 ± 0.64 a	2.46 ± 0.12 a

* Means followed by the same letter (a) in the same row are not significantly different according to ANOVA (Tukey’s test), *p* ≤ 0.05.

**Table 7:** Correlation coefficients obtained when experimental data are fitted to different models.

Sample	Correlation Coefficients (r^2^)			
	Zero-order	First-order	Higuchi	Korsmeyer–Peppas
A	0.6241	0.4783	0.8422	0.8524
B	0.7433	0.7319	0.9423	0.9819
C	0.7268	0.6628	0.9339	0.9601
D	0.7279	0.6600	0.9339	0.9601

**Table 8:** The diameter and intensity of the peaks in size distribution curves obtained for the samples of the dissolution medium during the in vitro drug release testing of the tablets A–D.

Time	15 min				6 h			
Peak	Peak 1		Peak 2		Peak 1		Peak 2	
Sample	D (nm)	I (%)	D (nm)	I (%)	D (nm)	I (%)	D (nm)	I (%)
A	73.4	100.0	/	/	147.4	100.0	/	/
B	210.2	96.3	5319.0	3.7	126.4	98.2	5236.0	1.8
C	389.6	100.0	/	/	140.1	100.0	/	/
D	165.5	94.1	5271.0	5.9	239.3	93.0	5007.0	7.0

D—Diameter; I—Intensity.

## Data Availability

All data generated during the research are included in this manuscript. Further details and support are available from the corresponding author on the request.
